# Proteoglycans in Cancer: Friends or Enemies? A Special Focus on Hepatocellular Carcinoma

**DOI:** 10.3390/cancers14081902

**Published:** 2022-04-09

**Authors:** Francesco Dituri, Gianluigi Gigante, Rosanna Scialpi, Serena Mancarella, Isabel Fabregat, Gianluigi Giannelli

**Affiliations:** 1 National Institute of Gastroenterology Saverio de Bellis, IRCCS Research Hospital, Castellana Grotte, 70013 Bari, Italy; gianluigi.gigante@irccsdebellis.it (G.G.); rosanna.scialpi@irccsdebellis.it (R.S.); serena.mancarella@irccsdebellis.it (S.M.); gianluigi.giannelli@irccsdebellis.it (G.G.); 2 Oncobell Program, Bellvitge Biomedical Research Institute (IDIBELL), CIBEREHD and University of Barcelona, L’Hospitalet de Llobregat, 08908 Barcelona, Spain; ifabregat@idibell.cat

**Keywords:** proteoglycans, cancer, HCC, tumor microenvironment

## Abstract

**Simple Summary:**

Proteoglycans affect multiple molecular and cellular processes during the progression of solid tumors with a highly desmoplastic stroma, such as HCC. Due to their role in enhancing or limiting the traits of cancer cells underlying their aggressiveness, such as proliferation, angiogenesis, epithelial to mesenchymal transition (EMT), and stemness, these macromolecules could be exploited as molecular targets or therapeutic agents. Proteoglycans, such as biglycan, versican, syndecan-1, glypican-3, and agrin, promote HCC cell proliferation, EMT, and angiogenesis, while endostatin and proteoglycan 4 were shown to impair cancer neovascularization or to enhance the sensitivity of HCC cells to drugs, such as sorafenib and regorafenib. Based on this evidence, interventional strategies involving the use of humanized monoclonal antibodies, T cells engineered with chimeric antigen receptors, or recombinant proteins mimicking potentially curative proteoglycans, are being employed or may be adopted in the near future for the treatment of HCC.

**Abstract:**

Proteoglycans are a class of highly glycosylated proteins expressed in virtually all tissues, which are localized within membranes, but more often in the pericellular space and extracellular matrix (ECM), and are involved in tissue homeostasis and remodeling of the stromal microenvironment during physiological and pathological processes, such as tissue regeneration, angiogenesis, and cancer. In general, proteoglycans can perform signaling activities and influence a range of physical, chemical, and biological tissue properties, including the diffusivity of small electrolytes and nutrients and the bioavailability of growth factors. While the dysregulated expression of some proteoglycans is observed in many cancers, whether they act as supporters or limiters of neoplastic progression is still a matter of controversy, as the tumor promoting or suppressive function of some proteoglycans is context dependent. The participation of multiple proteoglycans in organ regeneration (as demonstrated for the liver in hepatectomy mouse models) and in cancer suggests that these molecules actively influence cell growth and motility, thus contributing to key events that characterize neoplastic progression. In this review, we outline the main roles of proteoglycans in the physiology and pathology of cancers, with a special mention to hepatocellular carcinoma (HCC), highlighting the translational potential of proteoglycans as targets or therapeutic agents for the treatment of this disease.

## 1. Introduction

Proteoglycans are heavily glycosylated proteins consisting of a protein core covalently linked to one or more anionic glycosaminoglycans (GAGs) chains, which are ubiquitously represented in the animal extracellular matrix. Generally, their localization is variable and can involve the cell membrane, the pericellular space, or the extracellular interstitium. In some tissues, such as cartilage, proteoglycans represent the dominant component of the extracellular matrix. Being present in different tissues of multicellular animals, proteoglycans are likely to perform a great variety of functions. Some of these functions are structural, as they generally maintain the hydration state of the extracellular milieu, thus helping to redistribute the mechanical loads. In addition, proteoglycans act as molecular “sieves” to perform a size-dependent selection of permeable compounds that results in the exclusion of exceedingly heavy macromolecules [1,2]. In recent decades, more complex functions of proteoglycans have been described, such as the control of the activity of extracellular enzymes, the interaction with several growth factors (including FGFs, BMPs, Wnts, IGFs, etc.) and their related receptors, and the regulation of pathways involving these molecules. In tissue development and repair, proteoglycans may play a key role in controlling homeostatic gradients and the availability of potent growth factors, and it is therefore, it is easy to understand how they can be involved in disease and cancer [3].

Structurally, proteoglycans consist of a central core protein that covalently binds one or more GAG chains. GAGs are long, unbranched polysaccharide chains composed of repetitive disaccharide units, which, in turn, include one amino-monosaccharide and one often acid monosaccharide containing sulfate and/or carboxyl groups. These disaccharides can involve couplings such as the following: N-acetylglucosamine/glucuronic acid, N-acetylglucosamine/iduronic acid, N-acetylgalactosamine/glucuronic acid, N-acetylgalactosamine/iduronic acid, and N-acetylgalactosamine/iduronic acid galactose. Known GAGs are quite limited in number, and the most relevant include Heparan Sulfate (HS), Dermatan Sulfate (DS), Chondroitin Sulfate (CS), Hyaluronate (HA), and Keratan Sulfate (KS) [4]. Although the number of proteins that can be conjugated with GAGs to form proteoglycans is also small, the resulting macromolecules are classified into large families and are involved in a surprisingly wide range of processes [5]. Proteoglycans have been identified primarily as components of the extracellular matrix, although they can also be localized in the pericellular space, or within the cell membrane. Several GAGs and proteoglycans have been evidenced at the nuclear level. Although the nuclear localization of GAGs and proteoglycans has been controversial for years, studies using radiolabeled [35S] sulfate and confocal microscopy have helped to clarify this uncertainty [6,7]. It has been shown that heparan sulphate (HS) and several proteoglycans, especially heparan sulphate proteoglycans (HSPGs), can localize within the nucleus and interact with chromatin and multiple transcription factors to regulate cell cycle and gene expression [8]. For example, a shed form of syndecan 1 (sSDC1), released by cancer cells, can be harvested from extracellular medium by bone marrow-derived stromal cells and conveyed into the nucleus where it binds to and inhibits the function of histone acetyl transferase p300 [9]; moreover, heparan sulfates were found to prevent transcription factors, such as AP-1, SP-1, ETS-1, and nuclear factor κB, from interacting with their consensus DNA sequences in HepG2 cells of hepatocellular carcinoma (HCC) [10]. The mechanism that mediates nuclear transfer of proteoglycans is not fully elucidated, but probably requires partial cleavage of specific domains that would result in the delivery of GAGs into the nucleus [5]. Indeed, many proteoglycans undergo proteolytic cleavage. For example, syndecans are subjected to shedding by a variety of matrix proteinases (including metzincins), especially during physio-pathological processes such as wound healing, and cancer cell proliferation, migration, and invasion [11]. HSPGs are involved in the clathrin- and caveolin-independent endocytic degradation of many extracellular ligands, including cationic polymers, lipids, and polypeptides, by conveying them through lysosomal degradative pathways [12]. The activity of proteoglycans can be modulated by post-translational modification based on proteolytic cleavage operated by a variety of enzymes that release extra-, or intra-cellular fragments endowed with signaling activities. For example, sheddases are matrix metalloproteinases (MMPs) that can cleave membrane proteoglycans such as syndecans to release extracellular ectodomains, whereas heparanases have heparin sulphate as a substrate, and sulphatases cut 6-O sulfates from heparin sulfate GAGs chains. Soluble ectodomains can diffuse within the tissues of origin and paracrinally influence the behavior of neighboring cells. They are also often released into the bloodstream, thus becoming of some relevance as circulating biomarkers [13,14,15]. This level of control is reflected in the biological properties of cells in normal as well as cells under pathological conditions such as inflammation and cancer.

**Figure 1:**
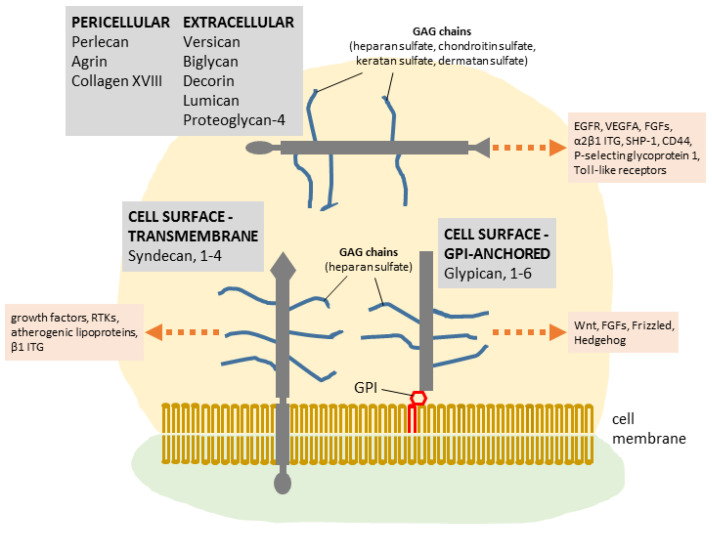
Proteoglycans generally localize in the cell membrane, pericellular, or extracellular space. Large complexes can form between extracellular proteoglycans and hyaluronan, which can coordinate several units of proteoglycans to form a highly hydrated gel-like matrix (not shown). Multiple interactions occurring between proteoglycans and extracellular ligands, or cell membrane receptors (shown as orange arrows) may modulate their downstream signaling effects. RTKs, receptor tyrosine kinases; ITG, integrins; GPI, glycosylphosphatidylinositol.

A number of growth factors carry binding sites for heparin, suggesting that proteoglycans may have a role in facilitating their signaling activities. Jiao and colleagues found that HSPGs are required in bone morphogenetic processes by virtue of their ability to bind bone morphogenetic protein BMP2 and permit its internalization into myoblasts, thus enabling BMP2-mediated osteoblast differentiation [16]. Heparan sulfate present on the cell surface is essential for the inter- and intracellular signaling pathways, as it interacts with and mediates the stimulatory function of multiple ligands, such as basic fibroblast growth factor (bFGF) [17,18,19]. Not surprisingly, HSPGs play a pivotal role in the development of invertebrates and vertebrates. Mutational alteration of the exostosin glycosyltransferase 1 and 2 (EXT1 and EXT2) genes, which encode for enzymes required for HS chain elongation, leads to lethality in mice and an autosomal hereditary disorder called multiple exostoses in humans [20,21,22]. Over the years, therefore, focus has shifted to embryonic stem cells and their potential for treating a range of diseases. Stem cells are relatively rich in proteoglycans and heparan sulfate (HS) has been shown to be essential for differentiation [23,24]. For example, embryonic stem cells derived from Ext1 deficient mice (Ext1−/−) that lack HS expression and localization at their outer surface, are incapable of differentiation but can be rescued by exogenous supplementation of heparin or HS [25]. These experiments underscore the essential nature of heparan sulfate for mammalian development, possibly because essential growth and differentiation factors require GAG interactions on the cell surface. A simplified scheme summarizing the structure, localization, and interaction of proteoglycans with different interactors is shown in Figure 1.

## 2. Proteoglycans Support Cancer Progression by Regulating Cancer Cell Aggressiveness, Angiogenesis, Stromal Microenvironment, and Inflammation

The function of proteoglycans has been long investigated in relation to the multiple properties of cancer, including dysregulated cancer cell growth, angiogenesis, and stromal microenvironment remodeling. Being characterized by various and versatile biochemical features that make them able to interact with both ligands and receptors, proteoglycans perform different functions in cancer, also facilitating downstream activities of several signaling pathways. Whether proteoglycans act as tumor promoting or suppressing factors is not unequivocally determined, as it depends on the type of proteoglycans, their post-translational processing forms, as well as the nature and stage of neoplasm [15]. Perlecan (heparin sulphate proteoglycan 2, HSPG2) is considered as a pro- or anti-angiogenic factor depending on the post-translational form in which it acts. Aviezer et al. found that perlecan can promote the development of vasculature by virtue of its ability to increase the affinity of the pro-angiogenic factor fibroblast growth factor (FGF)-2 for the FGF receptor on endothelial cells [26]. By contrast, the C-terminal domain V of perlecan, also called endorepellin, which is produced by perlecan cleavage by catepsin L, was found to be capable of acting as an anti-angiogenic factor. By forming a complex with vascular endothelial growth factor receptor 2 (VEGFR2) and integrin α2β1 on endothelial cells, endorepellin induces a downregulated of VEGFR2 signaling activities, thus eliciting angiogenesis blockage [27,28]. In addition, an alternative anti-angiogenetic path was described for endorepellin, wherein this proteoglycan derivative inhibits angiogenesis via a mechanism that induces authophagy in a VEGFR2-dependent but α2β1-independent manner [29]. Based on these assumptions, the anti-angiogenic properties of endorepellin may be exploited in the setting of novel therapeutic strategies against various solid tumors. Regardless of its role in vascular development, perlecan supports cancer progression in experimental settings. In a recent study, Vennin et al. demonstrated that pancreatic cancer cells with hyperactivated p53 can epigenetically reprogram cancer-associated fibroblasts (CAFs). These so-educated cells, via an NF-κB-dependent pathway, release perlecan, which, in turn, promotes local invasiveness, metastasis, and chemoresistance to gemcitabine and Abraxane in various in-vivo models of pancreatic cancer [30]. Syndecan-1 (SDC1) is a cell surface multifunctional proteoglycan that was found to exert a protective function against the development of colorectal cancer in a murine model of inflammation and carcinogenesis (chemically induced by administering azoxymethane + dextran sodium sulfate, AOM/DSS). In these animals, the genic depletion of SDC1 led to increased susceptibility to colitis-associated tumorigenesis and was associated with an increased local level of interleukin 6 (IL-6) and consistently with more activated status of its receptor STAT3, and of several tumor-promoting STAT3 downstream genes [31]. Using CD44(+) CD24(−/low) breast cancer stem cells (CSC) that recapitulate the aggressive phenotype of neoplastic cells represented in inflammatory breast cancer, Ibrahim and colleagues found that the expression of SDC1 promotes efficient in vitro CSCs 3D spheroid and colony formation by maintaining an activated status of Notch signaling. The effects of the activation of this pathway on CSCs aggressiveness are probably mediated by Notch-induced expression of IL-6, IL-8, gp130, Hey-1, and EGFR, and Akt phosphorylation [32]. Lumican is a small leucine-rich keratin sulphate proteoglycan that may enhance or limit tumorigenesis in a context-dependent mode. While lumican overexpression has been associated with poor prognosis in some malignancies, such as breast and pancreatic cancers, its tissue level was found to be increased in osteosarcoma and melanoma patients with better survival [33,34,35,36]. Confirmation of the anti-cancer properties of lumican came from studies demonstrating the capacity of proteoglycan to inhibit in vitro colony formation of melanoma cells transformed by oncogenes H-ras, v-K-ras, and v-src. Cytostatic effects of lumican have been associated with the induction of the cyclin-dependent kinase (CDK) inhibitor p21WAF1 [15,37,38,39,40].

Aberrant biosynthesis of GAGs occurring in some pathological circumstances, may play a role in increasing the risk of HCC. It was determined that the loss of the suppressor gene exostosin-like 2 (Extl2) results in increased production of GAGs acting as damage-associated molecular patterns (DAMPs). By activating Toll-like receptor 4 (TLR4), these GAGs contribute to the inflammatory state and consequently the propensity for HCC development, especially in the presence of underlying obesity and diabetes [41]. The biological activity of proteoglycans can be modulated by cleavage by enzymes that can release fragments with various functional significances. For example, SDC1, which is a membrane proteoglycan, can be processed by sheddases (such as matrix metalloproteinase 7 or 9) that produce extracellular as well as membrane and intracellular fragments [42]. The soluble extracellular fragment, called ectodomain, can diffuse away from the cell surface to signal at distant targets, while the residual product in the membrane acts to mediate cell-cell or cell-matrix communications. Importantly, shed ectodomains can be delivered into circulation, and their detection can have a clinical and prognostic value, as reported for the SDC1 ectodomain in lung cancer [43]. The heparan sulfate chains of proteoglycans can be cleaved by heparanase, an enzyme endowed with endo-glucuronidase activity, which generates proteoglycan products that are highly active in promoting cancer progression and the dissemination of carcinomas, sarcomas, and hematological malignancies. Consistently with its tumor-supporting activity, heparanase expression is upregulated in carcinomas, sarcomas, and hematological malignancies [44]. Furthermore, heparin sulfate can be processed by sulfatases, such as Sulf-1 and Sulf-2. These enzymes are highly overexpressed in pancreatic cancer and HCC and perform sulfate removal from the 6-O position of proteoglycan on the outer side of the cell surface. This event results in a reduced interaction affinity between heparan sulfate proteoglycan (HSPG) and Wnt ligand, which in turn can bind to frizzled receptors and activate downstream canonical Wnt pro-tumorigenic signaling. Contrariwise, Sulfs can also induce the disassembling of tumor-supporting complexes, such as that involving FGF-2, HSPG, and FGFR1 [45,46,47].

Remodeling processes involving proteoglycans can have important implications in pathology as they have been shown to increase the aggressiveness of some cancers (such as breast carcinoma) by enhancing angiogenesis and cell invasion [15,48]. Versican (VCAN) is a proteoglycan represented by multiple isoforms (V0, V1, V3, V4) that are often overexpressed in the stroma of some solid tumors, including colorectal cancer, pancreatic cancer, and HCC. Regarding these neoplasms, a positive association was found between the accumulation of this proteoglycan in cancerous tissue and a poorer prognosis [49,50,51]. VCAN is expressed and secreted by cancer-associated fibroblasts under specific stimuli, such as the transforming growth factor-β (TGFβ) [52,53,54]. The contribution of VCAN to cancer progression is controversial. For example, as far as V0 and V1 isoforms stimulate migration and proliferation of melanoma cells, V3 isoform can impair tumor growth, while favoring lung metastases in experimental models of melanoma [55,56]. A possible partially explanatory mechanism for this behavior may be that the V3 isoform binds to CD44 on the cell surface of melanoma cells, thus interfering with the interaction involving CD44 and the EGFR-ErbB2 complex, ultimately attenuating downstream oncogenic signals [57].

Various carcinomas are characterized by a highly desmoplastic stromal reaction, consisting of massive deposition of extracellular matrix proteoglycans that are implicated in the remodeling of the cancer stromal milieu and, consequently, influence the progression of disease. SDC1 exemplifies the impact that proteoglycans can have on the architecture of the cancer stromal microenvironment, which in turn influences the behavior of cancer cells. Yang et al. found that SDC1 expressed by human or murine mammary fibroblasts, in cooperation with integrin ανβ3, promotes the assembly of ECM fibers in parallel arrays, which in turn can favor directional migration and invasion of breast cancer cells [58,59].

Decorin is a member of the small leucine-rich proteoglycan that was early reported to attenuate gromerulonephritis by binding TGFβ and thus preventing the contribution of this cytokine to tissue damage due to excessive ECM deposition [60]. A similar effect due to the presence of this proteoglycan was reported in liver fibrosis/cirrhosis, where decorin is believed to “refrain” the pro-fibrogenic activity of TGFβ [61]. Decorin was reported to suppress tumor growth and angiogenesis. The anti-tumor and anti-angiogenic functions of this proteoglycan can be attributable to its capacity to bind to several growth factors and receptors (including the already mentioned TGFβ, PDGF, activin C, CTGF, EGFR, c-Met, IGF-IR, VEGFR2, TLR2, and TLR4), thus promoting their degradation or inactivating their downstream signaling activity. Furthermore, as decorin expression is downregulated in many aggressive carcinomas, its tissue expression score is being exploited as a prognostic predictor [62,63]. Similarly, the small leucine-rich proteoglycan osteoglycin (OGN) was recently reported to be down-regulated in colorectal cancer patients with a worse prognosis. OGN has the ability to reduce proliferation and invasion of colorectal cancer cells by binding EGFR and promoting its internalization, resulting in the impairment of pro-mitotic signaling and EMT driven by the EGFR/AKT/Zeb-1 axis [64].

**Figure 2:**
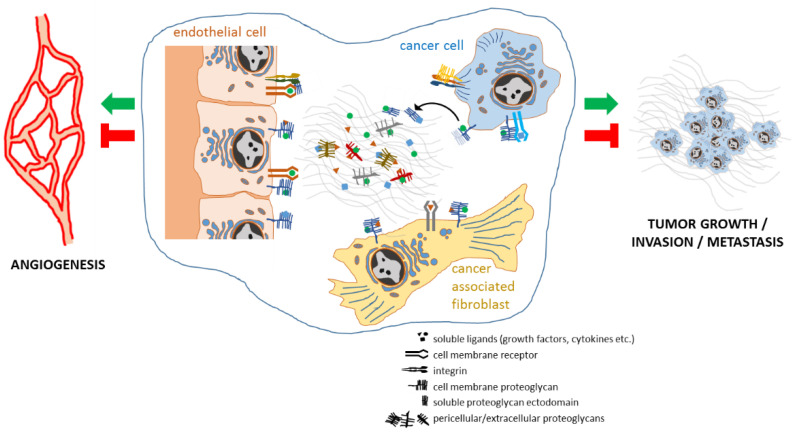
Localization of proteoglycans and some of their interactive partners within the stromal microenvironment of cancers. Three of the major cell type that produce and respond to proteoglycans stimulations are represented.

A simplified overview of the localization and functional relations between proteoglycans and cellular and other non-cellular elements of the cancer microenvironment is illustrated in Figure 2.

## 3. Proteoglycans and Liver Fibrosis, Cirrhosis and Non-Alcoholic Fatty Liver Disease

Cirrhosis, which is the most advanced stage of fibrotic processes that can occur during chronic liver disease, represents a major risk factor for the subsequent development of HCC, as suggested by the fact that 80–90% of HCC patients are cirrhotic. Liver fibrosis often results from the persistence of various etiologic factors (including HBV, HCV, alcohol, and NAFLD) that trigger and perpetuate liver parenchymal injury, inflammation, remodeling of the extracellular matrix, and regenerative nodules as compensatory mechanisms to replace necrotic hepatocytes [65,66]. Chronic hepatocyte damage and the continuous regenerative stimulus represent the main substrate for the development of fibrosis and subsequent cirrhosis. Dysregulated accumulation of extracellular matrix generally results from a concomitant increase in synthesis/deposition and a reduction in the degradation of the components of the hepatic extracellular matrix, including collagen proteins (collagen I, III, and IV), non-collagen proteins (fibronectin, laminin), and proteoglycans (such as those bearing HA, HS, and CS) [67]. Although a number of proteoglycans, along with other abnormally expressed ECM proteins, participate in building up the aberrant stromal environment of the chronically injured liver, their individual contributions can be supportive or counteractive to fibrosis. Parenchymal degeneration that occurs during the cirrhotic process is associated with changes in the expression and localization of some proteoglycans. Expression of VCAN was shown to be significantly upregulated in the livers of cirrhotic patients and from animal models of hepatic fibrosis. Silencing of VCANexpression in hepatic stellate cells (a major source of this proteoglycan) resulted in an attenuation of the pro-fibrotic phenotype of these cells, as evidenced by reduced expression of the myofibroblast marker alpha-smooth muscle actin (αSMA) and TGFβ, suggesting a role for VCAN as a modulator of liver fibrosis [68]. The immunostaining of SDC1 in liver specimens of patients with chronic liver disease was found to be positively correlated with the severity of fibrosis, thus suggesting its possible role as a driver of fibrogenesis [69]. Moreover, the immunoreaction of this proteoglycan switches from a faint basolateral localization in hepatocytes of a normal liver to a stronger circumferential appearance in cirrhotic samples [70]. Although this evidence suggests a causal link between SDC1 upregulation and the development of liver fibrosis, a recent study established that this proteoglycan could counteract the fibrogenic process. Regős and colleagues demonstrated that, during early stages of fibrogenesis experimentally induced in mice, a shed form of SDC1 that is produced as a result of the activity of MMP14 can interfere with the pro-fibrogenic activity of TGFβ by at least the following two modes: (1) by sequestering the cytokine, thus preventing its downstream effects; (2) by binding and downregulating thrombospondin-1, a glycoprotein that converts latent TGFβ into its active form [71,72]. Perlecan is physiologically present in the liver as a component of the basal membrane lining the peri-sinusoidal sub-endothelial space of Disse [73]. During fibrosis, hepatic stellate cells (HSCs) stand out as a major source of ECM molecules, including type XVIII collagen, perlecan, and, together with other cells (such as Kupffer cells), ECM remodeling enzymes (mainly MMP2, 3, and 9) [74,75]. Perlecan was found to localize in reactive bile ducts in patients with chronic cholestatic disease [76]. Despite this evidence, the actual contribution of this proteoglycan to the pathogenesis of this disease and, in general, of chronic liver disease is not yet fully elucidated. On the contrary, a compelling role as a modulator of hepatic fibrogenesis was demonstrated for decorin, as previously stated. Decorin was found to be produced by HSCs under TGFβ1 stimulus and appears histologically coexpressed and colocalized with this cytokine in a spectrum of liver diseases, including chronic hepatitis, fibrosis, and cirrhosis. This evidence suggests that decorin may have a protective action, offsetting pro-fibrotic TGFβ1 activity. To support this hypothesis, more rigorous approaches were employed both in vitro and in vivo, which clearly demonstrated that decorin can hinder the activity of TGFβ1 in inducing ECM protein synthesis and fibrosis. Further anti-fibrotic actions of this proteoglycan have been attributed to its ability to sequester receptors for growth factors, such as EGFR, IGFR, and Met, in addition to TGFβ [61,77,78].

Nonalcoholic fatty liver (NAFL) and nonalcoholic steatohepatitis (NASH) are conditions framed in the more general context of nonalcoholic fatty liver disease (NAFLD) that can arise in association with obesity, metabolic syndrome, and type 2 diabetes [79]. Some proteoglycans have proved promising as biomarkers of NAFLD. Serum endocan levels resulted significantly lower in patients with NAFLD than in healthy controls. Furthermore, serum values of this proteoglycan showed a negative correlation with body mass index in the same subjects [80]. Contrariwise, serum syndecan-1 levels were significantly increased in NAFLD patients compared to controls, even if no significant correlation between serum values and immunohistochemical score of related liver biopsies was found [81]. In a 2009 study by Charlton and colleagues, extensive mass spectrometry analysis of the hepatic proteome of liver samples encompassing the entire histologic spectrum of NAFLD was performed. Among all the proteins screened, lumican turned out to be overexpressed in progressive NAFLD [82]. Although evaluation of tissue or circulating levels of disease-specific proteoglycans may be advantageous for diagnostic purposes or for monitoring the disease stage, many aspects of the molecular mechanisms in which they are involved require further investigation.

## 4. Proteoglycans and Liver Regeneration

Liver regeneration usually occurs when pathological, surgical, or traumatic events lead to the depletion of hepatic mass. The capacity of the liver to replace the missing tissue relies on the compensatory proliferative expansion of quiescent hepatocytes adjacent to the lesion, which results in the restoration of its original size, architecture, and functional status [83,84]. A multitude of growth factors and cytokines ligands and receptors contribute to same extent to the reparative hepatocyte proliferation. These include tumor necrosis factor (TNF-α), interleukin-6 (IL-6), hepatic growth factor (HGF), epidermal growth factor (EGF), fibroblast growth factors (FGFs), vascular endothelial growth factor (VEGF), insulin-like growth factors (IGFs), Wnt proteins, Notch receptors and relative ligands (Notch1-4, Jagged, Delta), members of transforming growth factor β superfamily, including TGFβ, activins, and bone morphogenetic proteins (BMPs) [85]. Spatial and temporal patterns of deposition of different components of the extracellular matrix, including collagen, fibronectin, laminin, and proteoglycans (perlecan, decorin, chondroitin-sulphate, and heparin-sulphate), have been investigated in the livers of humans affected by chronic liver diseases or animal models subjected to partial hepatectomy or acute liver injury. In a 1996 study, Gallai et al. monitored the expression kinetics of syndecan, perlecan, fibroglycan, and decorin after partial hepatectomy in rats by Northern blot analysis. A strong early upregulation at 30 min in decorin expression was observed, followed by the first peaks of syndecan and perlecan at 2 and 4 h after hepatectomy. At 24 h post hepatectomy, all three of these proteoglycans had a high level of transcription at steady state. Contrary to the above-mentioned proteoglycans, fibroglycan expression dropped after partial hepatectomy, reaching a constant minimum level over time [86]. In another report, condroitin sulphate deposition was also detected within a few hours, reaching a peak level in regenerating tissue at 24 h after hepatectomy, followed by a gradual declination [87]. Increased collagen synthesis combined with reduced activity of cathepsin L, which generates endostatin by clivating collagens (such as collagen XVIII), were observed in rat livers within 7 days of hepatectomy [88,89]. This early evidence suggests that coordinated modulation of expression of specific proteoglycans may have a functional meaning in hepatic regeneration.

Glypican 3 (GPC3) is a cell surface HSPG that is highly expressed in embryonic tissues but not detectable in the normal liver [90]. The function of GPC3 was suspected to be relevant in liver regeneration, as a loss-of-function mutation of its related gene leads to a X-linked disorder called Simpson–Golabi–Behmel syndrome, which is characterized by the pre- and post-natal liver overgrowth and a considerable risk of developing embryonic tumors during childhood [91]. Liu et al. investigated its role in normal liver regeneration and hepatocyte proliferation. They found that GPC3 mRNA and protein levels begin to increase following partial hepatectomy, reaching a maximum and plateau at day 5, whereas the hepatocyte proliferation rate decreases in a temporally coordinated way. Consistently, in in vitro studies, GPC3 was found to prevent hepatocyte overgrowth, probably by a mechanism involving the interaction with the member of the tetraspanin family CD81, the expression of which results also elevated upon hepatectomy [92]. The same group has demonstrated that, when transgenically overexpressed in mice, GPC3 slows down hepatocyte proliferation and liver regeneration after hepatectomy [93].

Heparan sulfates (HS) are probably the major GAGs present on the surface of hepatocytes under normal conditions. Nevertheless, HSPGs expression increases during liver regeneration. Using [35S] sulfuric acid incorporation, Otsu et al. showed that, in the hepatic regeneration phase after hepatectomy, the synthesis of heparin sulfate proteoglycans, and to a lesser extent, of chondroitin/dermatan sulfate proteoglycans, increases up to 3–5 days and is temporally shifted compared to the stage of maximum mitosis that occurs 1–2 days following the surgical procedure [94]. However, Kimura et al. have reported that heparan sulfate appears just hours after hepatectomy, suggesting a possible role of this GAG as an initiator of hepatocyte proliferation [95].

In a 1996 study, Yada et al. reported changes in the pattern of expression of ECM components in hepatic tissue during rat liver regeneration after partial hepatectomy. In the regenerative phase, starting 24 h after partial hepatectomy, type I and III collagen displayed a more marked and continuous accumulation pattern throughout the hepatic lobule, while antibodies against type IV collagen, fibronectin, and perlecan yielded stronger immunoreactivity in sinusoid regions, compared to sham operated controls [87].

## 5. Proteoglycans Involved in HCC Progression

HCC is a leading cause of cancer-related mortality worldwide. The high inter- and intra-tumor heterogeneity due to the underlying pathology, on which HCC arises (frequently chronic inflammation and/or cirrhosis), associated with the poor knowledge of the molecular mechanisms underlying the progression of this neoplasm, makes it difficult to develop treatment strategies based on precision medicine approaches [96,97]. The complexity of the HCC microenvironment amply accounts for the difficulty of deciphering the functional significance of multiple interactions involving cellular elements, mainly cancer, stromal, endothelial, and immune cells, and an extraordinarily enriched extracellular milieu. GAGs and proteoglycans certainly play a significant role within this complexity.

There is a significant quantitative and qualitative change in hepatic GAGs during the development and progression of HCC. For example, the levels of chondroitin sulfate, low molecular weight GAGs, as well as nonsulfated and disulfated chondroitin sulfate disaccharide units increase, while heparan sulfate concertedly decreases during the worsening of HCC [98].

Heparan sulphate (HS) was not found to be differently expressed or significantly modified in total sulfation degree in HCC compared to normal liver tissue [99]. However, it has been shown that this GAG, in the form bound to the plasma membrane heparan sulfate proteoglycan syndecan-4 (SDC4), can bind and enhance the signaling activity of stromal derived factor-1 (SDF-1/CXCL12), resulting in increased growth and invasion of hepatoma cells in response to interaction between this ligand and its receptor, CXCR4 [100]. Syndecan-1 (SDC1) is a transmembrane proteoglycan, the expression of which is higher in cirrhotic than in HCC tissue. It was found that an extracellular segment of SDC1 can be shed from the cell surface and consistently detected in serum. Although circulating levels of SDC1 have been positively correlated with HCC progression, it is unclear to what extent this evidence reflects its actual contribution to the disease. SDC1 may affect key features of HCC progression, such as cell migration, EMT, and stemness. Indeed, it was shown that SDC1, in functionally coupling with TGFβ1, is required for HCC cells to undergo EMT in response to sphingosine-1-phosphate (S1P) and that SDC1 maintains the expression of cancer stemness markers CD13 and CD44 in tumor-spheres made from HCC cells. Nevertheless, how this proteoglycan influences cellular and molecular aspects of HCC progression in vivo is yet to be determined [101,102,103,104]. Among membrane-anchored proteoglycans, glypicans are of particular interest in liver neoplasms. One member of these proteoglycans, GPC3, has been extensively investigated and related to the development of HCC, for which it has a diagnostic and prognostic value [105]. GPC3 is virtually absent in the normal liver but is highly expressed in the tumor tissues of HCC patients and is detected at an elevated concentration in the serum of the same subjects, whereas it is less represented in the tissues of benign liver disorders [106,107]. More specifically, GPC3 is expressed only in embryonic tissues but reappears during malignant hepatocyte transformation as an oncofetal protein [108]. Shirakawa et al. found that higher levels of GPC3 in HCC tissues are correlated with a poorer prognosis [109,110]. A role for GPC3 in the cancerogenesis of HCC has been described in reference to its ability to promote epithelial-to-mesenchymal transition via ERK signaling and to stimulate cell proliferation through enhancing oncogenetic pathways such as the Wnt/Frizzled and IGF signaling axis [111,112,113]. Based on these results, the potential of GPC3 as an immunotherapeutic target in proteoglycan-overexpressing tumors is being tested in clinical trials [114]. Agrin, a pericellular HSPG present in the basal lamina of normal tissues, was found to be upregulated in cirrhotic and HCC microenvironments. Of interest, it appears to be localized in the proximity of bile ductules of cirrhotic livers and newly formed microvessels of malignant tumors [115]. Agrin expression was found to be positively related to the progression of HCC and was proposed as a prognostic marker of disease outcome [116]. Importantly, agrin was shown to be secreted by HCC cells, endothelial and hepatic stellate cells, and to generate oncogenic signaling that enhances cancer cell proliferation, migration, and EMT through a mechanism that is Arp2/3-dependent [117]. In addition, a neoangiogenetic function of this proteoglycan has been described in solid tumors, including HCC. More specifically, in mouse models of liver cancer, it was reported that a complex interaction involving agrin, LDL receptor-related protein 4 (Lrp4), β1 integrin, and focal adhesion kinase (FAK) mediates the adhesion of endothelial cells to cancer cells, thus promoting the sprouting of new microvessels, and that depletion of this proteoglycan results in suppressed tumor growth and metastasis to the lungs [118]. Endostatin is the C-terminal domain of Collagen XVIII and is released following elastase activity. This proteoglycan was found to counteract VEGF- and bFGF-induced endothelial cell proliferation and migration, besides promoting the apoptosis of these cells, thus ultimately acting as an anti-angiogenic factor [119,120,121]. Biglycan belongs to the small leucine-rich proteoglycans (SLRP) family and may have a relevant role in HCC progression as it can interact with surface receptors such as toll-like receptors (TLRs) 2 and 4, CD14, and CD44, which in turn influence processes such as production of inflammatory cytokines (especially in macrophages), autophagy, angiogenesis, cell growth, and migration, to eventually promote tumor progression or suppression [122,123]. Hyaluronate and VCAN have been found to promote tumor cell proliferation and metastatic capacity by binding to the same receptors [124,125]. The expression of VCAN and biglycan is upregulated by TGFβ in cells of mesenchymal origin both in normal and pathological conditions, such as HCC, wherein stromal cells, mainly cancer associated fibroblasts (CAFs), display a sustained secretion of extracellular matrix (ECM) proteins, including proteoglycans [53,126,127,128].

Extensive crosstalk between HCC cells and stromal cells is a requisite for productive tumor progression. This interaction is mediated by a plethora of structural ECM proteins and soluble factors, including hormones, growth factors, proteolytic enzymes, inflammatory cytokines, lipids, but also peptides and glycoproteins derived by proteolytic cleavage of membrane receptors and proteoglycans [129,130]. A large contribution to HCC ECM accumulation derives from the activity of CAFs. The mutual interaction between these stromal elements and epithelial tumor cells is believed to play a decisive role in the progression of HCC and, consequently, in the clinical progression of the disease [131,132]. CAFs can be phenotypically programmed by adjacent malignant cells and, in turn, increase the proliferation and spread of HCC cells, possibly through the secretion of several molecules, including extracellular matrix (ECM) proteins [133,134]. CAFs deliver ECM components including type I and III fibrillar collagen and non-collagen glycoproteins, such as fibronectin, laminin, hyaluronate, elastin and proteoglycans [135]. CAFs from ovarian cancer can secrete the VCAN in response to TGFβ. This proteoglycan, in turn, promotes the motility and invasion of ovarian cancer cells by a mechanism involving activation of the NF-κB signaling pathway and overexpression of matrix metalloproteinase-9 (MMP9) and CD44 [52]. It was also found that HCC CAFs increased VCANmRNA expression upon exposure to TGFβ [53]. VCAN (especially the versicanV1 isoform) has been involved in the metastatic progression of HCC. Specifically, this proteoglycan promotes the secretion of the chemokine (C-C motif) ligand 2 (CCL2) from HCC cells. CCL2 is a potent chemoattractive stimulus for macrophage infiltration within tumors, a process that turns out to be important as a promoter of dissemination [136]. More recently, Zhangyuan et al. found that expression of VersicanV1 is significantly increased in tumors of HCC patients and is related to worse prognosis. Importantly, the same authors describe an interaction between the VersicanV1 and the EGFR-PI3K-AKT axis, which, in turn, enhances the Warburg effect of HCC cells, ultimately leading to proliferation, invasion, and metastasis [50].

CD44 was already well known as a widely recognized receptor for several ligands, including hyaluronate, osteopontin, and matrix metalloproteinases [137]. In light of this recently acquired knowledge, it is increasingly regarded as a radical marker in HCC [138]. The overexpression of CD44 in this tumor is an early event during the onset of carcinogenesis, which is responsible for the acquisition of a phenotype resistant to senescence and the accumulation of mutations by the transforming hepatocytes [139]. It was found that HCC CAFs secrete proteoglycan 4 (PRG4), a high molecular weight proteoglycan previously characterized as an important constituent of the joints synovium, wherein it works as a lubricant factor to prevent frictional degeneration of cartilage [140]. Al-Sharif et al. reported that PRG4 can interact with CD44, in competition with hyaluronan that also binds this surface receptor. Moreover, these authors demonstrated that PRG4, by binding to CD44 can counteract CD44-dependent downstream signaling that leads to growth induced by pro-inflammatory interleukin-1β (IL-1b) stimulation [141]. We have recently demonstrated that PRG4, upon binding to CD44, interferes with the capacity of this receptor to mediate resistance to sorafenib and regorafenib, both drugs employed as HCC treatment options [53].

## 6. Proteoglycans as Circulating Biomarkers for the Detection and Staging of HCC

As with other HCC markers, the aberrant expression of specific proteoglycans in HCC tissues often results in their effusion into the bloodstream, thus providing the opportunity to evaluate their use as circulating biomarkers. Unfortunately, only a limited number of proteoglycans have proved reliable enough for early detection of HCC, recurrence, or assessment of staging.

A serum measurement of SDC1 proved useful for HCC detection and assessing the staging of the disease. By comparing patients with liver cirrhosis and with HCC, Metwaly et al. found that SDC1 levels were significantly increased in the sera of HCC subjects as compared with the cirrhotic group. Moreover, SDC1 serum levels also resulted positively correlated with the stage as assessed by the Barcelona-Clinic Liver Cancer (BCLC) staging system [102]. In another study, high circulating values of SDC1 and endocan were found to be significantly associated with an increased risk of relapse in patients with early HCC who had received radiofrequency ablation treatment [142].

Soluble GPC3 is detected in the serum of 40–53% of patients with HCC but is not present in the serum of healthy individuals [106]. In a meta-analysis study, measurement of serum GPC3 displayed a sensitivity and specificity of 55.1% and 97.0% in diagnosing early-stage HCC, respectively. The sensitivity increased up to 76% for tumors < 3 cm in size when GPC3 and alpha-fetoprotein were combined [143,144]. By quantifying serum GPC3 levels in patients with virus-related cirrhosis as a surveillance approach to predict or detect HCC, Caviglia and colleagues found moderate diagnostic accuracy (area under the curve, AUC = 0.637) for this proteoglycan [145]. However, a more powerful predictive approach based on the measurement of circulating biomarkers to assess the risk of developing HCC in nonalcoholic fatty liver disease (NAFLD) affected livers has been tuned through stratification of patients by age, gender, and the levels of protein induced by vitamin K absence or antagonist-II (PIVKA-II), GPC-3, and adiponectin (AUC = 0.948) [146].

## 7. Proteoglycans as Therapeutic Targets or Agents in HCC

Due to the dysregulated expression and involvement of some proteoglycans as supporters or antagonists in the progression of liver cancers, they are exploitable as potential targets or therapeutic tools. In particular, with regard to HSPGs, the expression of GPC3, perlecan, and agrin is increased, while that of SDC1 is reduced in HCC, compared to normal liver [147]. By binding to a large array of receptors and ligands, HSPGs are able to perform both structural and signaling functions in solid tumors, including HCC [47]. Some enzymes involved in the remodeling of the stromal microenvironment can hydrolyze HSPGs. These include MMP9, heparanase, and sulfatase-2 [148,149,150,151]. Hydrolysis of HSPGs substrates delivers at least the following three products: the above-mentioned SDC1 and GPC3, and fascin that can promote HCC cell invasion [152]. The inhibition of the activity of these proteoglycans is considered attractive as a therapeutic tool for the treatment of HCC. For example, the inhibitor of SDC1, synstatin, was proven to reduce the expression of pro-angiogenetic factors VEGF and FGF-2 through promoting the downregulation of αVβ3 integrin in thioacetamide-induced HCC in rats [153]. The interest in targeting GPC3 is heightened by the fact that this proteoglycan is expressed uniquely in HCC but not in the normal liver [106,154]. Monoclonal antibodies against GPC3 have been developed and show antitumor potential in diverse HCC models [155]. In particular, a fully humanized monoclonal antibody was found to evoke antibody-dependent cell-mediated cytotoxicity when administered in both in vitro and in vivo experimental models employing GPC3 positive HCC cells [156,157]. Moreover, administration of anti-GPC3 antibodies in a phase I clinical trial resulted in some tumor limiting capacity [158,159]. In a recent study by Li et al. (2020), engineered T cells that express chimeric antigen receptors (CARs) consisting of humanized antibodies targeting GPC3 were administered to mice bearing xenografted tumors obtained through injecting Hep3B and HepG2 HCC cells. Noteworthy, this treatment resulted in a complete ablation of GPC3 cells, likely via a mechanism involving perforin- and granzyme-mediated apoptosis or impaired Wnt signaling [160].

As far as some proteoglycans are being considered as targets due to their cancer-promoting activity, other proteoglycans turned out to represent possible pharmacological agents, as they were reported to play anti-angiogenic or drug-enhancing functions. The proteolytic fragment of the heparan sulfate proteoglycan collagen XVIII called endostatin was found to work as an anti-angiogenic factor, as it counteracts the action of VEGF and bFGF/FGF-2 [161,162,163]. To exploit the potential of endostatin as a novel therapeutic tool, a recombinant human form was developed (Endostar) [164]. This agent was demonstrated to inhibit endothelial HUVEC cell proliferation, migration, invasion, and tubulogenesis per se, or as a result of interaction with HCC cells in vitro, probably via the impairment of the Wnt/β-catenin signaling pathway [165,166]. Proteoglycan 4 (PRG4 or also called lubricin), a high molecular weight proteoglycan, is abundantly present in the synovium of joints, where it plays a role as a lubricant, thus contributing to maintaining their physiologic homeostatic state [167]. This proteoglycan turned out to mitigate the severity of osteoarthritis and other inflammatory or degenerative illnesses of joint cartilage, as demonstrated in studies using animal models, wherein it is exogenously administered in the diseased sites, or its expression is transgenically modulated [168,169]. PRG4 was recently demonstrated to impair the in vitro motility and invasion of breast cancer cells in response to TGFβ pro-migratory stimulation. More specifically, PRG4 competes with hyaluronan for binding to CD44, and once bound to this receptor, inhibits its downstream signaling. In addition, PRG4 inhibits hyaluronan biosynthesis and TGFβ-induced CD44 up-regulation [170]. It was recently found that PRG4 is expressed in liver and HCC tissues and is able to enhance sorafenib and regorafenib effectiveness in slowing down the in vitro proliferation of HCC cells expressing the PRG4 receptor CD44 [53]. Based on this evidence, PRG4, like endostatin, may be of interest as a novel, naturally occurring factor in the treatment of HCC.

**Figure 3:**
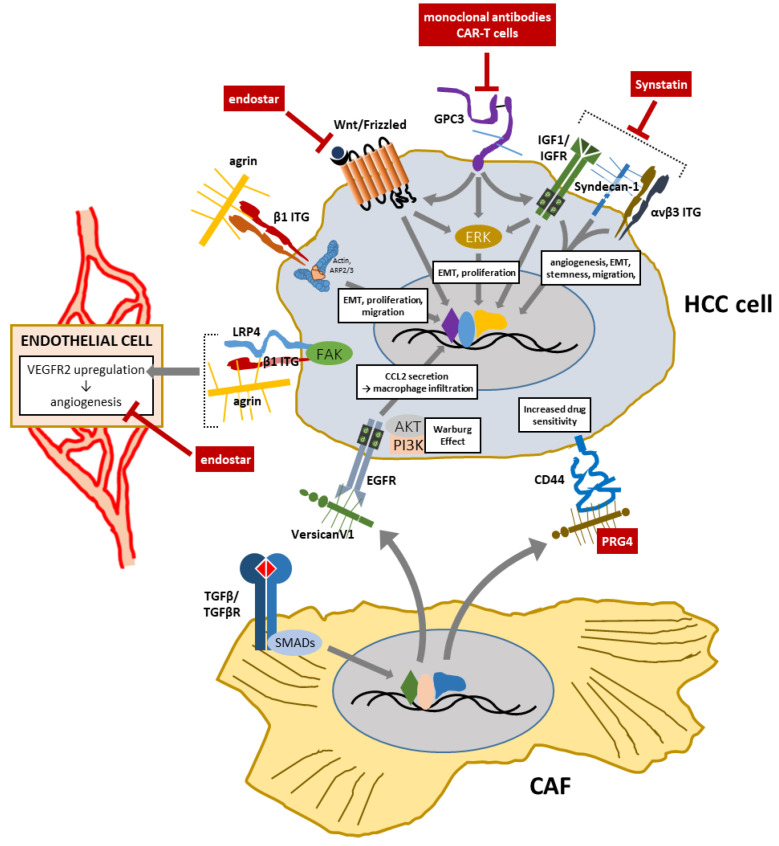
Contributions of major proteoglycans to HCC progression at cell level and their potential/current employment as molecular targets or therapeutic agents.

An overview of treatment options (potential, tested in animal models, or in use in clinical practice) that involve targeting proteoglycans as targets or curative agents for the treatment of HCC is shown in Table 1 and Figure 3.

## 8. Conclusions

The field of knowledge of proteoglycans and GAGs in physiology and cancer is constantly evolving, as novel biochemical properties of these macromolecules are being uncovered. Due to their involvement in all the cellular processes participating in disease progression, such as angiogenesis, growth factor-mediated signaling, drug resistance, along with beneficial or detrimental effects of their upregulation or deficiency, proteoglycans can be intended as targets for anti-tumor therapies or curative agents. HCC is a tumor where a typically dense and abundant extracellular matrix is often observed. Unsurprisingly, proteoglycans are noteworthy as HCC progression players, as they are highly expressed and affect HCC cell proliferation, invasion, and angiogenesis. Obviously, further research will be necessary to clarify several aspects of the interactive biochemical network occurring between proteoglycans and other factors and define the pattern of expression and function of proteoglycans within the HCC tumor microenvironment, ultimately, to identify specific patient subsets and, consequently, better address the design of proteoglycans-based or proteoglycans-targeting therapies.

So far, a number of biophysical and biochemical properties of GAGs and proteoglycans in various physiological and pathological contexts have been investigated across a large body of research covering at least three decades. This characterization has revealed the multitude of functions that proteoglycans perform by complexing with soluble ligands and/or cell membrane receptors (growth factor receptors, integrins, etc.). As an integral part of the cancer stromal microenvironment, proteoglycans have been intensely studied regarding their involvement in the progression of solid tumors, especially HCC, since this is a cancer with an extracellular milieu usually enriched with ECM molecules. The expression of some proteoglycans, or the presence of specific fragments derived from enzymatic proteoglycans cleavage, such as the processed products of HSPG, SDC1, GPC3 and fascin, was found to be related to the progression of HCC. Contrariwise, the presence in HCC of other proteolytic products of proteoglycans or full-length proteoglycans, such as endostatin and PRG4, is potentially beneficial due to their anti-angiogenic and drug-enhancing abilities. The development of therapies based on compounds to target detrimental proteoglycans or on recombinant proteoglycans that limit tumor progression represents a concrete opportunity to improve the prognostic perspective of HCC patients. The main pitfalls arising when designing novel inhibitors, as for many small-molecule drugs, lie in the risk of general toxicity and/or low selectivity. Humanized monoclonal antibodies, such as those targeting GPC3, may offer some advantages more than simply blocking the critical functional domain of the proteoglycan. Indeed, GPC3-targeted Chimeric Antigen Receptor (CAR)-T cells-based therapies have been registered for HCC and related phase I and II trials are ongoing [171]. Employing proteoglycans in native, recombinant, or tampered forms for curative purposes in HCC is a reasonably pursuable route. An example is the aforementioned recombinant endostatin, which is currently being tested in two clinical trials (according to ClinicalTrials.gov, accessed on 7 April 2022). PRG4 is a possible novel agent to be addressed by virtue of preliminary evidence that can enhance sorafenib and regorafenib effectiveness in inhibiting HCC cell proliferation [53]. A major issue with using PRG4 as an anti-tumor agent stems from the very high molecular weight of this molecule (>300 kDa). Therefore, its bioavailability would be seriously impaired if the mode of administration employed the typical routes. A possible strategy to bypass this problem would consist of processing the entire molecule to obtain a much smaller fragment while still retaining the domains or portions that are critical for the biological effects of interest. As the interaction of PRG4 with its best-known receptor, CD44, that is expressed in most invasive HCC cell lines, is required for PRG4-mediated enhancement of drug effects, the mapping of CD44-binding sites on PRG4 would be a preliminary step to guide the design of specific PRG4-derived bioactive fragments.

## Figures and Tables

**Table 1:** Physiopathological and clinical value of proteoglycans in HCC.

Proteoglycan	Relevance in HCC	Therapeutic Options	References
GPC3	- Is upregulated in HCC compared to normal liver tissue (prognostic value) - Promotes EMT (via ERK signaling) - Promotes cell proliferation (via Wnt/Frizzled and IGF signaling)	Monoclonal targeting antibodies	[106,154,155,156,157,158,159,160]
	CAR-T cells		
HS proteoglycan collagen XVIII	- Endostatin (proteolytic fragment): anti-angiogenic	Endostar (recombinant endostatin): inhibits endothelial cell proliferation, migration, invasion, tubulogenesis	[87,88,119,120,121,161,162,163,164,165,166]
Proteoglycan 4 (PRG4)	- Variable expression in HCC tissues - Expression correlated with better prognosis - Enhances drug’s effectiveness	PRG4 fragments binding CD44 (?)	[53]
SDC1	- Cooperates with TGFβ to promote S1P-induced EMT - Maintains the expression of cancer stemness markers (CD13, CD44)	Synstatin (inhibitor): counteracts angiogenesis (reduces the expression of VEGF and FGF-2 in a HCC rat model)	[101,102,103,104,155]
VersicanV1	- Expression positively correlated with worse prognosis - Promotes the secretion of macrophage-attracting CCL2 - Enhances Warburg effect, cell proliferation and invasion	None to date	[50,136]
Agrin	- Expression positively correlated with progression (prognostic value) - Enhances cancer cell proliferation, migration, and EMT (via Arp2/3-dependent signaling) - Promotes sprouting of new microvessels (via interaction with Lrp4, β1 ITG and FAK)	None to date	[115,116,117,118]
